# Factors to Consider When Assessing the Diagnostic Accuracy of Synovial Leukocyte Count in Periprosthetic Joint Infection

**DOI:** 10.7150/jbji.34854

**Published:** 2019-08-02

**Authors:** Karsten D Ottink, Carol Strahm, Anneke Muller-Kobold, Parham Sendi, Marjan Wouthuyzen-Bakker

**Affiliations:** 1 Department of Orthopaedics, University of Groningen, University Medical Centre Groningen, the Netherlands; 2 Division of Infectious Diseases and Hospital Epidemiology, Cantonal Hospital St. Gallen, Switzerland; 3 Department of Laboratory Medicine, University of Groningen, University Medical Centre Groningen, The Netherlands; 4 Department of Infectious Diseases and Hospital Epidemiology, University Hospital Basel, University Basel, Basel, Switzerland; 5 Department of Orthopaedics and Traumatology, University Hospital Basel, Basel, Switzerland.; 6 Institute for Infectious Diseases, University of Bern, Bern, Switzerland.; 7 Department of Medical Microbiology and Infection Prevention, University of Groningen, University Medical Centre Groningen, The Netherlands

**Keywords:** periprosthetic joint infection, synovial fluid, diagnosis, white blood cell count, polymorphonuclear percentage

## Abstract

Synovial white blood cell (WBC) count and the percentage of polymorphonuclear leucocytes (PMN%) is one of the diagnostic criteria to diagnose a periprosthetic joint infection (PJI). Although the test is widely available, the diagnostic accuracy of proposed cut-off levels are influenced by several factors, such as: the affected joint, co-morbid conditions, the causative microorganism and the gathering and processing of samples in the laboratory. In this narrative review we provide an overview on how and to what extent these factors can affect the synovial WBC count and PMN% in synovial fluid.

## Introduction

The diagnosis of a periprosthetic joint infection (PJI) is challenging especially for chronic PJIs. Because there is no single and absolute test to confirm or exclude infection 1-7, patients' history, physical examination, joint specific x-ray or other types of imaging, histology, culture and inflammatory markers should all be taken into consideration for diagnosis. It is critical to distinguish between septic and aseptic failure preoperatively, as arthroplasty exchange of a missed PJI leads to subsequent failure 8,9. Criteria for the diagnosis of PJI have been proposed by the Musculoskeletal Infection Society (MSIS) and the International Consensus Meeting (ICM) 10-12. One of them includes the synovial white blood cell (WBC > 3000cells/µL) count and its proportion of polymorphonuclear cells (PMN% > 70). The test can be performed relatively easily in the preoperative diagnostic work-up. Its sensitivity, specificity and diagnostic accuracy depends on the threshold that is applied. Factors such as joint site, co-morbid conditions, the causative microorganism and the duration of infection, in addition to pre-analytical laboratory variables, may influence the optimal cut-off result 13. This narrative review provides an overview of these factors and its possible influence on the threshold and accuracy of the WBC count and PMN% in synovial fluid for PJIs.

## Search strategy and selection criteria

We identified references for this narrative review through searches of PubMed and Embase electronic databases by using the following combinations of terms (alternative terms grouped in parentheses): (“synovial*” OR “joint fluid” OR “synovial fluid”) AND (“white blood cell” OR “WBC” OR “leucocyte” OR “polymorphonuclear” OR “PMN”) AND (“count*” OR “differentiation” OR “analysis”)). We included publications from January 1966 to December 2018. The title and abstract were screened to identify the references applicable to the different research questions of this review, after which the full text articles were evaluated.

## Historical perspective: from native to prosthetic joints

Synovial fluid analysis has traditionally been considered in native joints to differentiate between non-inflammatory (typically with a WBC count of < 2,000 cells/µL and PMN% of < 25) and inflammatory arthritis^14^,^15^. In a systematic review on native septic arthritis, synovial WBC count of >25,000 cells/µL, >50,000 cells/µL and >100,000 cells/µL revealed a sensitivity of 77%, 62% and 29% and a specificity of 73%, 92% and 99%, respectively^16^.

The diagnostic use of joint aspiration before revision arthroplasty was first described by Duff *et al*. 17 in 1996. The authors retrospectively reviewed the preoperative aspiration results of 43 knees between 1985 and 1995. They investigated microbiological growth but did not report results of WBC count analyses. Spangehl *et al*. 18 prospectively analysed the WBC count in synovial fluid in 183 cases during revision of total hip arthroplasties (THAs) from 1994 to 1996. The authors considered it a positive finding if the WBC count was > 50,000 cells/μL or the PMN% was > 80% (i.e.; cut-offs used for native septic arthritis). They reported a good specificity of 99% (96%-100%) but a poor sensitivity of 36% (19%-56%). Kersey *et al*. 19 reported a mean WBC count of 782 cells/μL (11 to 7,200/μL) and a PMN% of 13 in 77 total knee arthroplasties (TKAs) undergoing revision for 'aseptic failure' from 1992 to 1997. The authors calculated that a WBC count of <2,000/μL and a PMN% of < 50 had a 98.3% negative predictive value for excluding infection.

In 2003, Mason et al. 20 demonstrated an important finding for the diagnosis of PJI. The specificity for diagnosing PJI with a WBC count cut-off value of 50,000 cells/μL is similar to the specificity of using a value of 2,500 cells/μL. They retrospectively reviewed preoperative aspiration data of 86 knees from 1986 to 1997 and calculated a specificity of 95% and sensitivity of 98% for diagnosing PJI when using a WBC count cut-off value of 2,500 cells/μL combined with a PMN% of > 60. After Trampuz *et al*. 21 (1,700 cells/μL for knee PJI) and Schinsky *et al*. 22 (3,000 to 4,200 cells/μL for hip PJI) reported similar findings in 2004 and 2008, respectively, it was apparent that considerably lower WBC count cut-off values should be used for the diagnosis of PJI than for the diagnosis of native septic arthritis.

## The confusion about the units

The observation that lower cut-off levels for the synovial WBC count were necessary in prosthetic joints than in native joints (i.e., more than 1 log), together with the use of different units, can cause some confusion in the nomenclature. The frequently quoted unit is cells/μL 23, equivalent to cells/mm^3^, 10^6^ cells/ L or cells/10^-3^cm^3^. The units may vary between institutions, depending on laboratory practice. Thus, attention to detail is required when converting the results from one unit to another to prevent errors 24.

## Factors potentially influencing synovial WBC count thresholds

Synovial WBC count cut-off values ranging from 1,000 to 5,000 cells/μL for the diagnosis of chronic hip and knee PJI have been published. Subsequently, there is an ongoing discussion about the optimal cut-off value. For simplicity, surgeons and physicians like to use a single optimal cut-off value. However, several variables may influence the final result, as discussed in the following paragraphs.

### Preanalytical steps

- USE OF LOCAL ANAESTHETICS: The influence of local anaesthetics on synovial fluid WBC has not been studied. However, local anaesthetics and related compounds may inhibit the growth of bacteria, presumably because of the acidity of most anaesthetic compounds. Therefore, local anaesthetics prior to joint puncture is discouraged 25.
- PRIOR USE OF ANTIBIOTICS: Lower WBC counts and PMN% in synovial fluid have been found in patients treated with antimicrobial therapy than in patients without antibiotic therapy 26. Therefore, antibiotics should be withheld in suspected chronic PJIs until a proper diagnostic work-up has been performed.
- VOLUME: The optimal synovial fluid volume for analyses of WBC count is typically 1 mL or more. However, depending on the analytical procedure, smaller volumes are possible (e.g., 250 µL). It is important to interact closely with laboratory experts to clarify these details, since there are inter-institutional differences in standard operating procedures between laboratories.
- DRY TAP: In case of a punctio sicca (“dry tap”), injection and re-aspiration of sodium chloride may be helpful to obtain bacterial culture. However, WBC analysis is not reliable anymore and should not be performed. Of note, dry tap does not exclude an infection, and the frequency of dry taps may vary between joints. In a study by Zahar *et al*. 27, a complete synovial fluid analysis was available in only 64% of all studied patients with a hip or knee arthroplasty joint due to a limited amount of synovial fluid, a clotted specimen or a bloody aspirate.
- CLOTTING: To prevent clotting, synovial fluid should be collected in ethylenediaminetetraacetate (EDTA) tubes. The tube should be turned upside down several times to mix the EDTA with the synovial fluid. Mucous samples are - due to its increased viscosity - difficult to pipet. The viscosity may also form a problem for automated cell analysers in aspirating a sufficient amount of fluid. Pre-treatment of the sample with hyaluronidase reduces the viscosity of synovial fluid and improves the accuracy of cytometric analysis, irrespective of whether optical or automated techniques are used 28. Of note, an EDTA tube is not preferred for crystal examination.
- BLOODY ASPIRATE: In the case of an aspirate with a high number of red blood cells (RBCs), it is possible to calculate an approximation of corrected values for the synovial WBC count by using a mathematical model and the serum WBC count 29. The adjusted synovial WBC count can be calculated with the following formula: synovial WBC _adjusted_ = synovial WBC _observed_ - [(WBC _blood_ / RBC _blood_) × RBC _synovial fluid_] 30. Discussion of the results with a laboratory expert is advised in these cases.
- TRANSPORT: Ex vivo cell lysis may occur during prolonged transport. Rapid transport of the sample to the laboratory with swift analysis of the material may prevent cell lysis. The optimal time interval has not been investigated for patients with PJI. We recommend analysis within 1 hour, preferentially within 30 minutes, of aspiration.
- LABORATORY CELL COUNTING: Studies have demonstrated that standardized automated counting of WBCs in synovial fluid is more accurate and efficient than manual counting 31-33. Manual counting of WBCs in synovial fluid results in an inter-observer variance of > 20% 28,31,34. In case of frank pus or metal on metal arthroplasty, the sample consists of high numbers of necrotic and apoptotic leucocytes. These cellular debris may result in false results in automated systems. In such a scenario, samples should be manually counted by a laboratory technician.

### Joint site

The location of the affected joint has an effect on the optimal threshold of the WBC count and the PMN%. A possible hypothesis for this is because of the differences in joint space/volume, synovial lining and vascularization. All of these factors theoretically may be influence the concentration of WBC and PMN% in a joint.

- HIP: Several studies investigated WBC count and PMN% in PJI diagnosis of the hip 27,35-45. These studies indicate an optimal cut-off for the WBC count of 3,000 cells/μL and a PMN% of 65. This cut-off value was supported by a recent meta-analysis that demonstrated subsequent sensitivity and specificity of 90% (95% confidence interval [CI] 0.828-0.938) and 86% (95% CI 0.704-0.939), respectively, for the WBC count and of 88% (95% CI 0.818-0.927) and 82% (95% CI 0.759-0.870), respectively, for PMN%.
- KNEE: We reviewed 15 studies that investigated the WBC count and PMN% in PJI diagnosis of the knee 27,35,37,38,42,46-55. In a meta-analysis performed by De Fine *et al.*, a higher diagnostic accuracy was observed in TKA PJI compared to THA PJI (Table 1) when using a threshold of 3,000 cells/µL and a PMN% of >65 55. It should be noted that due to the lack of an adequate number of clinical studies specifically involving TKAs, the precise assessment of the optimal cut-off threshold for TKA PJI was precluded in the meta-analysis 55. Zahar *et al*. 27 performed a single-centre retrospective study that included 179 patients with TKA. The authors calculated an optimal cut-off threshold for the WBC count of 1,630 cells/μL with a sensitivity of 84% and specificity of 82%. The optimal cut-off for PMN% was set at 61, resulting in a sensitivity of 80% and a specificity of 77% 27. Together with the previously mentioned data of Mason *et al.* 20 and Trampuz *et al*. 21, the results tended to demonstrate lower cut-off levels in knee joints than in hip joints.
- SHOULDER: Only a few studies have evaluated the diagnostic accuracy of the WBC count and PMN% in shoulder arthroplasties, and all of these studies comprised small cohorts 56,57. Recently, Strahm *et al*. 58 analysed 19 failed shoulder implants in which a WBC count and PMN% was performed prior to revision surgery. Sixteen of these cases (84%) were diagnosed with infection; the optimal synovial WBC count threshold was 12,200 cells/μL (sensitivity 92% and specificity 100%) and the optimal PMN% threshold was 54 (sensitivity 100% and specificity 75%). This higher threshold for infection in shoulders compared with hips and knees was also observed in previous reports 59. These data indicate that a different threshold should be applied for shoulder arthroplasties, but larger studies are needed to confirm these findings.
- ANKLE AND ELBOW: The joint volume of the ankle and elbow is limited. This is particularly the case after previous surgery and the presence scar tissue. Consequently, synovial fluid can rarely be aspirated and no published data is available about the diagnostic accuracy of WBC count and PMN% in the ankle 60 or elbow 61.

### Co-morbid conditions

It is conceivable that co-morbid conditions and immunosuppressive drugs have an influence on the production and function of leucocytes.

- INFLAMMATORY ARTHRITIS: Because an inflammatory arthritis causes an increase in synovial WBC 62, it is reasonable to assume a different treshold in a rheumatic than in a non-rheumatic joint with PJI. However, the study by Cipriano *et al*. 37 indicated that there is no clear difference in thresholds for the WBC count and PMN% for diagnosing a PJI in patients with and without inflammatory arthritis. Indeed, the meta-analysis performed by Qu *et al*. 63, in which the authors compared 8 studies that excluded patients with inflammatory arthritis (n=2102) with 6 studies that included patients with inflammatory arthritis (n=598), demonstrates no differences in the diagnostic accuracy of synovial fluid WBC count and PMN% between groups. Because of the high heterogeneity of the included studies, the authors were not able to determine the optimal cut-off values in the meta-analysis 63. In a multicentre study performed by Shohat *et al*. 64 that included 1,220 patients undergoing revision arthroplasty, thresholds for WBC count associated with PJI in patients with and without inflammatory arthritis were 2,533 and 2,683 cells/μL, respectively, and 73 and 72 for PMN%. Notably, in this analysis, the sensitivity was approximately 8% higher and the specificity approximately 15% lower in patients with inflammatory arthritis than in patients without inflammatory arthritis. However, the same thresholds were applied for both patient categories. The use of immunosuppressive drugs were not taken into account in these studies.
- METAL-ON-METAL (MoM) PROSTHESIS: In MoM prosthesis, metal wear from either the trunnion between the stem and the head or from the joint line of the THA, causes free metal particles. These particles may cause lymphocyte-dominated vasculitis-associated lesions (ALVAL) 40. Both the free metal particles and the subsequent inflammatory reaction make the synovial fluid analysis more difficult to perform, and also, to interpret. The diagnosis of PJI in patients with failed MoM bearings and/or corrosion reactions in hip arthroplasties is challenging 65. The clinical presentation can mimic a PJI, and aspiration of joint fluid may demonstrate pus 66. Hence, analysis of synovial fluid sample is not possible in these cases, because of the presence of metal or amorphous material, fragmented cells and/or clots. A manual count may identify the presence of metal or amorphous material. Also, the synovial WBC and PMN% are false positive for infection in these cases 66. However, the proportion of these misleading results in patients with MoM is difficult to assess 40,44. Several studies demonstrated a relatively high diagnostic accuracy of synovial WBC count and PMN% for the diagnosis of PJI in patients with MoM. In a study including 102 MoM THAs, the optimal cut-off threshold for the WBC count in diagnosing PJI was 3,111 cells/μL (sensitivity 94%, specificity 86%), and thus, similar to non-MoM prostheses. The optimal cut-off for PMN% was 85 with a sensitivity of 82% and specificity of 87% 66.
- CRYSTAL-INDUCED ARTHRITIS IN ARTHROPLASTY: Crystal-induced arthritis (gout, pseudogout) in a periprosthetic joint is a rare phenomenon 67-70. Analysis for crystal disease should always be included in the diagnostic workup of PJI, in particular in the case of predisposing factors for (pseudo) gout, such as alcohol abuse, renal failure or the use of diuretics, especially in patients with an acute presentation of arthritis 71. Crystal-induced arthritis may present with clinical manifestations that are identical to those of a PJI 72, and high WBC counts are reported in cases of acute gout (e.g.; up to 500,000 cells/µL) 73. It should be noted that the presence of crystal arthritis does not rule out a concurrent PJI. Patients with crystal-induced diseases may have a incidence of PJIs than patients without gout or pseudogout 74,75.

## Causative microorganism and bacterial inoculum

The microorganisms causing PJI largely depend on the type of infection (acute or chronic). Most diagnostic studies that evaluate the WBC count do not describe the cultured microorganisms. It is reasonable to assume that low-grade microorganisms, such as *Cutibacterium acnes*, cause a different or limited immunological response in comparison to that of more virulent microorganisms, like *Staphylococcus aureus, Streptococcus* species or Gram negatives 76,77. Indeed, it has been demonstrated that the sensitivity of several (synovial) biomarkers in *C. acnes* PJIs are moderate to poor 77,78. Recently, Kheir *et al*. 79 described the accuracy of several diagnostic tests per causative microorganism in 549 chronic PJIs and 653 aseptic revisions. The optimal cut-off for the WBC count was around 2,700-3,000 cells/μL irrespective of the causative microorganism, but appeared to be lower for *Enterococcus* species (optimal cut-off of around 1,700 cells/μL). The same applied to the PMN%, which was ~65 for most microorganisms, but ~58 for *Enterococcus* species. Notably, only 14 PJIs caused by enterococci were included in the study and infections caused by *C. acnes* were not reported in this cohort.

## Currently applied thresholds for WBC count in synovial fluid

Different institutions have different practices regarding the use of one cut-off level for all joints or the use of joint-specific cut-off levels. In the latter case, providers frequently use WBC count cut-offs of 1,100 to 1,700 cells/µL for knee PJI and 3,000 to 4,200 for hip PJI 27,53,55. Renz *et al*. 80 suggested >2,000 cells/µL and >70% PMN for both joint sites and demonstrated a sensitivity of 85.5% (95% CI 75.6-92.6) and a specificity of 92.3% (95% CI 86.3-96.3). In a meta-analysis, a cut-off threshold for WBC count of >3,000 cells/μL (irrespective of joint site) was calculated to have the highest sensitivity of 89% (95% CI 0.86-0.91) and specificity of 86% (95% CI 0.80-0.90) 55. A cut-off threshold for PMN% of >65 was calculated to have the highest sensitivity of 89% (95% CI 0.82-0.93) and specificity of 86% (95% CI 0.77-0.92) 81. A recent study by Zahar *et al*. 27 solely analysed patients with a chronically painful hip and knee arthroplasty; data on the WBC count was available in 337 cases. The proposed cut-off in this study was in accordance with the previously proposed thresholds for infection of >3,000 cells/μL and a PMN% of 65. During the second International Consensus Meeting on Musculoskeletal Infections in Philadelphia, the agreed threshold for WBC count was >3,000 cells/μL and a PMN% of 70%, although a cut-off of 80% was validated in a cohort of 684 infected cases and 820 aseptic revisions for the latter 82,83. The discrepancies in proposed cut-offs between studies may be due to differences in the type of infections included (acute / chronic) and/or how the control groups without an infection are defined (e.g., exclusion or inclusion of coinflammatory conditions, metallosis).

## Conclusion

The synovial WBC count result is only one of several diagnostic results that may lead to a definite diagnosis of PJI. A threshold of > 3,000 cells/μL and a PMN% of > 70 has been recently suggested 84. Most studies, however, have suggested WBC count cut-off values ranging from 1,100 to 4,200 cells/μL and 60% to 89% for PMN% for the diagnosis of PJI which may be due to differences in included infections (acute and chronic) and the defined control group.

In order to improve the diagnostic accuracy providers should pay attention to pre-analytic steps, the joint site, comorbidities and conditions that can mimic PJI.

The vast majority of studies on the subject of this review stem from chronic hip and knee PJI. A lower WBC count cut-off than 3000 cells/μL may be applied for knees to diagnose a PJI. The synovial WBC count in inflammatory conditions such crystal-induced arthritis and metallosis may return false-positive results, but its diagnostic accuracy does not increase when providing a higher cut-off value.

For a single patient, it is important to take these variables into account, and, from a clinical point of view, PJI should be suspected when the cell count result falls above a “range” of cell count values instead of a precise cut-off value.

## Figures and Tables

**Table 1:** Synovial fluid analysis in THA vs TKA according to the meta-analysis of De Fine *et al*. 55.

**WBC count 3,000 cells/µL**	**Sensitivity**	**95% CI**	**Specificity**	**95% CI**
THA	0.89	0.83-0.93	0.86	0.72-0.93
TKA	0.90	0.77-0.96	0.94	0.63-0.99
**PMN% > 65**	**Sensitivity**	**95% CI**	**Specificity**	**95% CI**
THA	0.88	0.82-0.93	0.82	0.75-0.88
TKA	0.93	0.80-0.98	0.90	0.76-0.97

## Abbreviations

CI: confidence interval
EDTA: ethylenediaminetetraacetate
ICM: International Consensus Meeting
MoM: metal-on-metal
MSIS: Musculoskeletal Infection Society
PJI: periprosthetic joint infection
PMN%: percentage of polymorphonuclear leucocytes
RBC: red blood cell
THA: total hip arthroplasty
TKA: total knee arthroplasty
WBC: white blood cell
